# Adjustment to an Appropriate Bedtime Improves Nocturia in Older Adults: A Crossover Study

**DOI:** 10.1111/iju.70068

**Published:** 2025-04-26

**Authors:** Yoshinaga Okumura, Sou Nobukawa, Tomoaki Ishibashi, Tetsuya Takahashi, Masaya Seki, So Inamura, Minekatsu Taga, Masato Fukushima, Hirotaka Kosaka, Osamu Yokoyama, Naoki Terada

**Affiliations:** ^1^ Department of Urology, Faculty of Medicine University of Fukui Fukui Japan; ^2^ Department of Information Engineering Chiba Institute of Technology Chiba Japan; ^3^ Department of Psychiatry, Faculty of Medicine University of Fukui Fukui Japan

**Keywords:** chronotherapy, gerontology, nocturia, nocturnal polyuria, sleep disturbance

## Abstract

**Purpose:**

We examined whether nocturia could be improved by adhering to an appropriate bedtime as determined using a wearable device that measures sleep–wake activity.

**Materials and Methods:**

We enrolled patients aged 65 years or older with nocturia at our institution and associated hospital, for whom medical therapy was either not indicated or ineffective. We conducted a prospective crossover comparative study with alternate 4‐week intervention and non‐intervention periods with a 2‐week washout period. During the intervention, participants were instructed to go to bed at a personalized bedtime determined via the steepest descent method, using the data of bedtime and mid‐wake time from an Actiwatch Spectrum (Philips Respironics). A frequency volume chart was administered before and after each period. Sleep quality was evaluated using the Pittsburgh sleep quality index (PSQI).

**Results:**

The analysis included 24 patients enrolled for the study. The mean age was 79.7 years. The mean bedtime changed from 21:30 to 22:11 after intervention (*p* < 0.01). During the intervention and non‐intervention periods, the mean changes were as follows: nocturnal urinary frequency (−0.90 vs. −0.01 times, *p* < 0.01), nocturnal urine volume (NUV) (−105.6 vs. +4.4 mL, *p* = 0.04), hours to undisturbed sleep (HUS) (62.8 vs. 12.7 min, *p* < 0.01), NUV per hour during HUS (−28.4 vs. −0.17 mL/h, *p* = 0.04), and PSQI scores (−2.4 vs. 1.2, *p* = 0.022).

**Conclusions:**

In older patients with nocturia, going to bed at appropriate times, determined using a wearable device that measures sleep–wake activity, might potentially prolong HUS, decrease NUV, specifically NUV/h during HUS, and consequently improve nocturia and sleep quality.

**Abbreviations**
CI: confidence interval
HUS: hours to undisturbed sleep
IPSS: International Prostate Symptom Score
LUTS: lower urinary tract symptoms
NBC: nocturnal bladder capacity
NUF: nocturnal urinary frequency
NUV: nocturnal urine volume
NUV/h: nocturnal urine volume per hour
OABSS: overactive bladder symptom score
PSQI: Pittsburgh sleep quality index
SD: standard deviation

## 1 Introduction

The International Continence Society defines nocturia as the number of times an individual passes urine during their main sleep period, from the time they have fallen asleep up to the intention to rise from that period [1]. Nocturia by itself is associated with physical distress, daytime fatigue, and decreased quality of life [2]. Additionally, it is correlated with a wide variety of organic diseases and psychiatric disorders [3, 4, 5] as well as an increased risk of falls and hip fractures in older adults [6]. It is also associated with an increased risk of all‐cause morbidity and mortality [7].

The primary causes of nocturia include nocturnal polyuria, reduced functional bladder capacity, and sleep disorders. Of the three major causes of nocturia, sleep disturbance has a bidirectional relationship with nocturia [8]. Epidemiological studies have consistently shown that nocturia is the predominant cause of sleep disturbances across all age demographics, with its prevalence escalating with advancing age [2, 3, 9]. However, there are limited reports of prospective clinical trials suggesting that treating sleep disorders can lead to improvements in nocturia.

A common feature of aging is the tendency to adopt an earlier bedtime [10], often earlier than desired [11]. As a result, older adults sleep for a longer period, become shallow sleepers, and are easily awakened by a slight urge to urinate, which eventually leads to a longer mid‐wake duration. We hypothesized that adjusting bedtime improves nocturia in older adults. In this study, an appropriate bedtime was determined using data from a wearable device that measures sleep–wake activity, and the impact of adhering to these personalized bedtimes on nocturia symptoms was evaluated.

## 2 Methods

### 2.1 Participants

This study included patients who visited our hospital and affiliated institutes with nocturia as their chief complaint. The inclusion criteria were as follows: waking up more than once per night to urinate; persistent nocturia despite at least 12 weeks of standard urological treatment; lack of available medications under the Japanese Ministry of Health, Labor and Welfare's guidelines for appropriate use of public medical insurance; age 65 or older; ability to answer various questions; accurately record frequency‐volume charts; ability to correctly use a wearable device and written informed consent from the patient or a family member after a thorough explanation of study participation. The exclusion criteria included urinary retention, night shift work, and dementia.

The sample size was calculated using G*Power (ver. 3.1.9.4) [12] for a repeated‐measures analysis of variance based on improvements in NUF. A sample size of 24 participants was determined with the following parameters: Cohen's effect size of 0.25, *α* error of 0.05, and power of 0.8. Based on previous reports, we chose a moderate effect size of 0.25 according to Cohen's effect size criterion [13], as it represents a meaningful clinical difference in NUF—a reduction of 0.5 from 2.5 times per night—sufficient to justify implementation in practice [7, 14].

**FIGURE 1:**
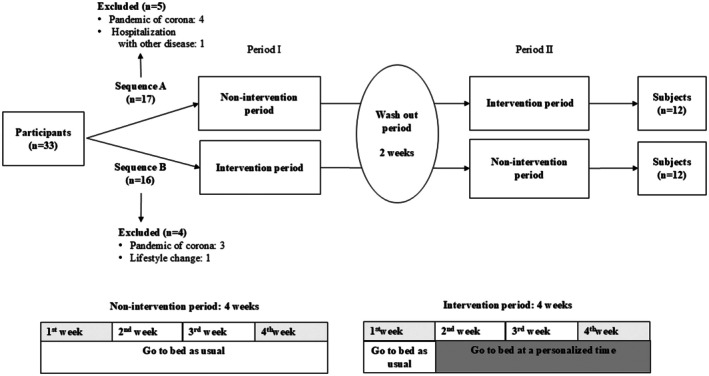
Flow chart of the study. Participants recorded their sleep–wake time using Actiwatch Spectrum, FVC, and questionnaires (PSQI, OABSS, and IPSS) during the first and fourth weeks of each period. Participants went to bed at a personalized bedtime, which was determined based on the data from the first week of the intervention period. FVC, frequency volume chart; IPSS, international prostate symptom score; OABSS, overactive bladder symptom score; PSQI, Pittsburgh sleep quality index; QOL, quality of life.

During April 2021 and December 2023, 33 participants were enrolled and alternately assigned to Sequence A and Sequence B groups in order of entry. Seven patients withdrew because of the difficulty in going to the hospital due to the coronavirus disease outbreak, one participant was hospitalized because of another disease, and another participant had a lifestyle change. Thus, the final analyses were performed with a total of 24 participants: 12 participants each from Sequences A and B (Figure 1).

### 2.2 Procedure to Determine Personalized Bedtime

To achieve a personally adjusted bedtime, we used a wrist‐wearable device, Actiwatch Spectrum (Actiwatch; Philips Respironics, Murrysville, PA, USA). The participants wore the device on their wrists, which captured their body movements using an acceleration sensor that determined their sleep and wake states, thus providing the participants with bedtimes, wake‐up times, sleep latency onsets, and mid‐wake durations. Based on the bedtime and overnight mid‐wake duration over 1 week, we estimated a correction in the amount of time required for bedtime to approach the optimal bedtime to achieve the minimum mid‐wake duration using the steepest descent method, which is an algorithm that searches for the minimum value of a function based only on its slope. Based on the bedtime (*X*) and mid‐wake time ($f\left(X\right)$) a linear relationship (corresponding to $\frac{df\left(X\right)}{d\;X}$) of the dependence of the mid‐wake time on bedtime is obtained. From this relationship, the amount of change of bedtime for approaching the optimal bedtime is determined. Specifically, the corrected bedtime $\hat{X}$ to be indicated for the participant was given by $\hat{X}=X-\alpha \frac{df\left(X\right)}{d\;X}$, where X is the average bedtime obtained during 1 week, $f\left(X\right)$ is a function of mid‐wake duration as bedtime, and $\frac{df\left(X\right)}{d\;X}$ is the gradient of the mid‐wake duration against bedtime. We adjusted the coefficient *α* = 150 to achieve $-90<\alpha \frac{df\left(X\right)}{d\;X}$< 90 min corresponding to one sleep cycle.

### 2.3 Study Design

We adopted an open‐label, multicenter, quasi‐randomized, crossover design with alternate 4‐week intervention and non‐intervention periods and a 2‐week washout period (Figure 1). Participants were alternately assigned to Sequences A (non‐intervention period—washout period—intervention period) and B (intervention period—washout period—non‐intervention period) in the order of entry. Participants wore an Actiwatch Spectrum in the first and last week. During the non‐intervention period, they went to bed at their usual bedtime. During the intervention period, a personalized bedtime was determined by the data obtained from the Actiwatch in the first week, and participants went to bed at the determined time during the last 3 weeks.

Participants were asked to follow certain instructions: (1) go to bed within 15 min of a specified bedtime as consistently as possible; (2) turn off the TV and any lights; (3) do not operate devices that emit light in bed, such as smartphones; and (4) avoid daytime naps as much as possible. The drug treatment was not quit and the fluid intake was not restricted during the study periods. A frequency‐volume chart spanning at least 3 days was administered before and after the intervention and non‐intervention periods. The PSQI, OABSS, and IPSS were assessed before and after both periods.

### 2.4 Statistics

The primary endpoint was the change in NUF. The secondary endpoints were changes in NUV, NUV/h, nocturnal bladder capacity, mid‐wake time, sleep‐onset latency, HUS, NUV until HUS, NUV/h until HUS, PSQI, OABSS, and IPSS between the intervention and non‐intervention periods. The outcomes were shown as mean ± SD. The differences were evaluated using a two‐way repeated‐measures analysis. Significance tests were based on two‐sided *α* = 0.05 (two‐sided 95% CIs). Correlations were tested using Spearman's rank test. Statistical analyses were performed using SPSS version 28.0 (IBM Corp., Armonk, NY, USA).

## 3 Results

Table 1 shows the background of the 24 patients in this study. Participants included 17 men and seven women. The mean ± SD ages were 79.7 ± 5.6 years. The comorbidities of LUTS were nocturnal polyuria in 17 patients, benign prostatic hyperplasia in 11 patients, overactive bladder in nine patients, and neurogenic bladder in two patients. The medications used for LUTS were α1 blockers in 11 patients, β3 agonists in eight patients, anticholinergic agents in five patients, vasopressin in three patients, 5α reductase inhibitors in two patients, and bethanechol in two patients.

**TABLE 1:** Patient's background.

	Total (*n* = 24)	Sequence A (*n* = 12)	Sequence B (*n* = 12)	*p*
Female/Male	7/17	3/9	4/8	—
Age (years)	79.7 ± 5.6	79.5 ± 5.4	80.0 ± 5.9	0.86
Body weight (kg)	61.7 ± 7.9	61.1 ± 6.2	62.3 ± 89.2	0.89
BMI (kg/m^2^)	23.8 ± 2.6	22.7 ± 2.3	24.9 ± 2.5	0.07
Comorbidity				
Hypertension	12	7	5	0.41
Diabetes mellitus	2	1	1	0.76
Hyperlipidemia	2	2	0	0.24
Chronic heart failure	2	1	1	0.76
Chronic kidney disease	1	0	1	0.50
Comorbidity of LUTS				
Nocturnal polyuria	17	9	8	0.65
Benign prostatic hyperplasia	11	7	4	0.21
Overactive bladder	9	5	4	0.50
Neurogenic bladder	2	1	1	0.76
Medication for LUTS				
α1 blocker	11	7	4	0.21
β3 agonist	8	5	3	0.33
Anticholinergic	5	1	4	0.16
Vasopressin	3	1	2	0.50
5α reductase inhibitor	2	1	1	0.76
Bethanechol	2	1	1	0.76

Abbreviations: BMI, body mass index; LUTS, lower urinary tract symptom.

For each participant, the optimal bedtime was determined using the algorithm. Figure S1 illustrates a typical example of the weekly relationship between the bedtime (*X*) and mid‐wake time ($f\left(X\right)$) data for a week for one participant. The mean bedtime was 21:35, and the mean mid‐wake time was 59.9 min. As a result, the slope $\frac{df\left(X\right)}{d\;X}$ was −0.44, and the optimal bedtime was $\hat{X}$= 21:35–150 × −0.44 = 22:41. The participants were asked to go to bed at their ideal bedtime during the intervention period. Table 2 shows the results for each parameter before and after the intervention and non‐intervention periods. In the intervention period, the bedtime significantly shifted to a later time (from 21:31 to 22:13, *p* < 0.01). In contrast, the wake‐up times did not significantly change (from 6:06 to 6:09, *p* = 0.93). The mid‐wake duration significantly decreased (from 71.3 to 55.9 min, *p* = 0.05), and the sleep onset latency significantly decreased (from 7.4 to 4.4 min, *p* = 0.02). The NUF significantly decreased (from 3.9 times to 3.0 times, *p* = 0.02) and the NUF index significantly decreased (from 39.3% to 32.7%, *p* < 0.01). The HUS significantly increased (from 2.4 to 3.4 h, *p* < 0.01) and the NUV/h during HUS significantly decreased (from 94.7 to 66.1 mL/h, *p* = 0.04). These results indicated that going to bed at an appropriate time determined by the data from a wearable device decreased NUF, increased HUS and decreased NUV/h during HUS. The OABSS significantly decreased (from 5.4 to 4.0, *p* = 0.05), and IPSS significantly decreased (from 10.6 to 8.4, *p* = 0.05), indicating that the LUTS was improved. PSQI significantly decreased (from 7.6 to 5.4, *p* < 0.01), indicating that the sleep quality was significantly improved. None of these parameters significantly changed in the non‐intervention period.

**TABLE 2:** Outcomes before and after the intervention and non‐intervention periods.

	Non‐intervention period (*n*=24)		*p*	Intervention period (*n*=24)		*p*
	Pre	Post		Pre	Post	
Bedtime	21:28 ± 0:54	21:27 ± 1:01	0.83	21:31 ± 0:51	22:13 ± 0:50	**< 0.01** *
Wake‐up time	6:14 ± 0:53	6:08 ± 0:48	0.51	6:06 ± 0:50	6:09 ± 0:42	0.93
Mid‐wake duration (min)	78.4 ± 58.6	63.7 ± 44.9	0.28	71.3 ± 51.0	55.9 ± 43.6	0.05*
Sleep onset latency (min)	7.4 ± 8.3	6.3 ± 8.3	0.68	7.4 ± 6.1	4.4 ± 6.4	**0.03** *
NUF (times)	3.7 ± 1.2	3.7 ± 1.1	0.99	3.9 ± 1.3	3.0 ± 1.3	**0.02** *
NUV (mL)	670.4 ± 450.8	674.7 ± 349.6	0.7	707.1 ± 375.2	601.5 ± 380.2	0.1
NBC (mL)	182.4 ± 82.0	188.2 ± 80.3	0.86	186.5 ± 84.2	207.1 ± 82.0	0.3
NUF index (%)	38.1 ± 8.6	39.1 ± 8.3	0.85	39.3 ± 9.9	32.7 ± 11.0	**< 0.01** *
NUV index (%)	46.6 ± 16.5	45.3 ± 11.7	0.88	44.1 ± 12.3	41.3 ± 14.8	0.43
NBC index (%)	57.9 ± 13.3	56.4 ± 7.3	0.91	54.6 ± 7.8	59.5 ± 8.5	0.07
HUS (h)	2.6 ± 0.9	2.8 ± 1.1	0.41	2.4 ± 0.9	3.4 ± 1.3	**< 0.01** *
NUV during HUS (mL)	199.9 ± 94.0	210.9 ± 95.0	0.48	199.3 ± 83.7	201.4 ± 75.3	0.81
NUV/h during HUS (mL/h)	85.9 ± 42.3	85.8 ± 50.5	0.8	94.7 ± 52.9	66.1 ± 34.2	**0.04** *
OABSS	4.2 ± 2.1	4.7 ± 2.6	0.1	5.4 ± 2.6	4.0 ± 2.5	0.05*
IPSS	9.3 ± 3.5	9.2 ± 3.7	0.85	10.6 ± 6.5	8.4 ± 6.6	0.05*
PSQI	7.9 ± 3.1	7.4 ± 3.6	0.37	7.6 ± 2.9	5.4 ± 3.2	**< 0.01** *

*Note:* Data are presented as mean (SD).

Abbreviations: HUS, hours to undisturbed sleep; IPSS, International Prostate Symptom Score; NBC, nocturnal bladder capacity; NUF, nocturnal urinary frequency; NUV, nocturnal urine volume; NUV/h, nocturnal urine volume per hour; OABSS, overactive bladder symptom score; PSQI, Pittsburgh sleep quality index.

* *p*‐values with *p* < 0.05 are represented in bold text.

**FIGURE 2:**
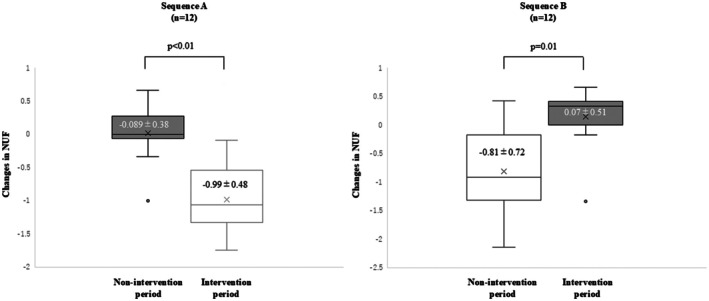
Differences between the changes in NUF. The difference in NUF during the non‐intervention period (−0.089 ± 0.38) and the intervention period (−0.99 ± 0.48) was significantly different (*p* < 0.001) in Sequence A (*n* = 12). The difference in NUF during the intervention period (−0.81 ± 0.72) and the non‐intervention period (0.07 ± 0.51) was significantly different (*p* = 0.01) in Sequence B (*n* = 12). CI, confidence interval; NUF, nocturnal urinary frequency.

Changes in NUF, the primary endpoint of this study, in non‐intervention and intervention periods in Sequence A and during the intervention and non‐intervention periods in Sequence B are shown in Figure 2. In Sequence A, the mean ± SD changes in NUF were − 0.09 ± 0.38 during the non‐intervention period and −0.99 ± 0.48 during the intervention period (*p* < 0.01). In Sequence B, the mean ± SD changes in NUF were − 0.81 ± 0.72 during the intervention period and 0.07 ± 0.51 during the non‐intervention period (*p* = 0.01). The changes in NUF were compared between the intervention and non‐intervention periods including both Sequences A and B. As shown in Table 3, the changes in NUF during the intervention period were significantly larger than those during the non‐intervention period (−0.90 times vs. −0.01 times, *p* < 0.01). During the intervention period, the correlation between the changes in bedtime and the changes in NUF was evaluated. As shown in Figure S2, the changes in NUF significantly correlated with changes in bedtime (*r* = −0.58, *p* = 0.003). These results indicated that the efficacy of the intervention to decrease NUF was higher for the patients with larger differences between the actual bedtime and the optimal bedtime.

**TABLE 3:** Outcomes of changes in the secondary endpoints.

	Non‐intervention period (*n*=24)	Intervention period (*n*=24)	*p* value
Changes in NUF (times)	−0.01 ± 0.46	−0.9 ± 0.62	< 0.01*
Changes in NUV (mL)	4.4 ± 161.0	−105.6 ± 255.1	0.04*
Changes in NBC (mL)	9.0 ± 44.2	20.6 ± 55.1	0.53
Changes in mid‐wake duration (min)	−14.7 ± 50.1	−15.2 ± 12.0	0.96
Changes in sleep onset latency (min)	−1.1 ± 10.6	−3.0 ± 8.5	0.57
Changes in HUS (min)	12.7 ± 58.7	62.8 ± 72.0	< 0.01*
Changes in NUV during HUS (mL)	11.0 ± 56.0	2.1 ± 56.5	0.6
Changes in NUV/h during HUS (mL/h)	−0.17 ± 40.8	−28.4 ± 48.0	0.04*
Changes in OABSS	0.5 ± 1.4	−1.4 ± 4.1	0.69
Changes in IPSS	1.0 ± 2.1	−2.0 ± 9.2	0.62
Changes in PSQI	1.2 ± 2.7	−2.4 ± 3.3	0.02*

Abbreviations: HUS, hours of undisturbed sleep; IPSS, International Prostate Symptom Score; NBC, nocturnal bladder capacity;NUV, nocturnal urine volume; NUV/h, nocturnal urine volume per hour; OABSS, overactive bladder symptom score; PSQI, Pittsburgh sleep quality index.

* *p* values with *p* < 0.05 are represented in bold text.

Table 3 also shows the changes of other parameters during the non‐intervention and intervention periods. The changes in NUV (−105.6 mL vs. 4.4 mL, *p* = 0.04), HUS (62.8 min vs. 12.7 min, *p* = 0.01) and NUV/h during HUS (−28.4 mL/h vs. −0.17 mL/h, *p* = 0.04) were significantly larger during the intervention period than the non‐intervention period. The changes in PSQI were also significantly larger during the intervention period than the non‐intervention period (−2.4 vs. 1.2, *p* = 0.02). These results suggested that the urine production levels after bedtime were decreased by the intervention, leading to an improved sleep quality. On the other hand, the changes in NBC, OABSS, and IPSS were not significantly different. These results suggested that bladder function was not improved by the intervention.

## 4 Discussion

Behavioral therapies such as exercise, diet, or fluid restriction are considered to be effective and standard treatment options for nocturia. In this study, we examined the efficacy of another behavioral therapy for nocturia, which is going to bed at an appropriate personalized time. We found that the NUF was significantly decreased in participants with nocturia when they were instructed to go to bed at a personalized optimal bedtime determined by our algorithm using the Actiwatch Spectrum. In addition, the HUS increased, the NUV decreased, especially in NUV/h during HUS, and the sleep quality improved. These findings suggest that adhering to this personalized schedule may be a promising behavioral therapy for nocturia.

Inappropriate sleep habits among older individuals are believed to play a significant role in nocturia. Older adults usually tend to go to bed earlier than their ideal bedtime [10, 11], which, in turn, leads to longer total sleep; however, they spend less time in the deeper stages of sleep [15]. They are also more likely to be awakened by signals from the stretch receptors in the bladder wall that a young adult might sleep through [16]. Tyagi et al. observed that older patients with insomnia treated with four simple behavioral treatments had significantly improved NUF compared with the information‐only controls [14, 17]. Khademi et al. suggested that personalized models using actigraphy could predict sleep parameters more accurately than generalized models, making them potentially useful for sleep health assessments and screening for sleep disorders [18]. Chiang et al. reported that personalized health behavior recommendations derived from wearable devices, which recorded physical activity data and sleep data, significantly improved participants' blood pressure [19]. Based on these findings, we hypothesized that proposing personalized sleep behavior patterns using data obtained from actigraphy could lead to improvements in nocturia. Therefore, in the present study, we considered methods for providing more personalized sleep behavioral treatment, and, after conducting multiple simulations, we devised a method to calculate the optimal bedtime using a mathematical approach that applies the steepest descent method. As a result, we successfully achieved significant improvements in both nocturia and sleep quality. Although restriction of fluid intake is important for improving nocturnal polyuria, we did not impose fluid restrictions or monitoring in this study to maintain a natural setting and enhance future clinical applicability.

The steepest descent method, which we used to determine the optimal bedtime, is one of the most basic algorithms for finding the minimum value of a function based on its slope. Based on various simulations conducted using data from prior volunteer studies, we decided to use this method in our research, taking into account the number of data points obtained and the burden on participants in terms of feasibility. The value of coefficient α was also determined through these simulations, and from an ethical standpoint, we set the adjusted bedtime not to exceed ±90 min. In this study, we used a wearable device that detects body movements to define mid‐wake time, which we regarded as an indicator of sleep quality [18, 19]. Ideally, brainwave measurements would be optimal for assessing sleep quality; however, measuring brainwaves at home for a week is anticipated to be challenging. Therefore, from a feasibility perspective, we chose to use an Actiwatch Spectrum to address that issue.

We previously reported that zolpidem, a non‐benzodiazepine hypnotic, dose‐dependently reduces urine output in vasopressin V2 receptor deficient rats [20]. We also demonstrated that melatonin decreases urine output and increases bladder capacity via the GABAergic system in rats [21]. In this study, the NUV significantly decreased beyond the effect of the shortened sleep time, but there was almost no increase in bladder capacity at night. Consequently, we hypothesized that older patients with nocturia might discover that modifying inappropriately long sleep durations could lead to improvements in sleep quality and to prolong HUS. This modification could potentially enhance melatonin secretion in the brain, subsequently resulting in a reduction of NUV, specifically the NUV/h during HUS.

To the best of our knowledge, this is the first study to verify the effectiveness of adjustment to an appropriate bedtime and regular sleep habits for nocturia. Nevertheless, this study also has some limitations. First, we determined the coefficient α of the formula of the steepest descent method to be 150 for the 1‐week data of bedtime and mid‐wake times from a previous simulation. Further verification is needed to determine if the value of α is appropriate. Second, although hormones such as melatonin or vasopressin should be associated with changes in NUV, these parameters were not evaluated in this study. We would like to measure them in future studies. Third, this study included only Japanese participants. Finally, we focused on older patients with nocturia and their sleep habits; this method can, however, still be used for younger patients with nocturia and sleep disturbance, and will be explored in future studies. Further studies are needed to establish a standard behavioral therapy for patients with nocturia and inappropriate bedtimes.

In conclusion, older patients with nocturia tended to go to bed earlier than their optimal bedtime. Going to bed at appropriate times determined by a wearable device that measures sleep–wake activity might significantly prolong HUS, reduce NUV, specifically the NUV/h during HUS, and consequently improve nocturia and sleep quality.

## Author Contributions

**Yoshinaga Okumura:** conceptualization, methodology, data curation, investigation, validation, formal analysis, funding acquisition, visualization, project administration, resources, writing – original draft, writing – review and editing. **Sou Nobukawa:** conceptualization, methodology, data curation, formal analysis. **Tomoaki Ishibashi:** conceptualization, methodology, data curation, formal analysis. **Tetsuya Takahashi:** conceptualization, methodology, data curation, investigation. **Masaya Seki:** investigation. **So Inamura:** investigation. **Minekatsu Taga:** investigation. **Masato Fukushima:** investigation. **Hirotaka Kosaka:** supervision. **Osamu Yokoyama:** supervision. **Naoki Terada:** supervision, writing – review and editing, writing – original draft.

## Disclosure

Registry and the registration no. of the study/trial: This study was registered with University hospital Medical Information Network (UMIN000051078).

## Ethics Statement

This study was approved by the Research Ethics Committee of the University of Fukui (reference number: 20210001) and was performed in accordance with the Declaration of Helsinki.

## Consent

Written informed consent was obtained from all participants.

## Conflicts of Interest

The authors received a grant from the Japanese Society of Geriatric Urology in 2021 for this research. Naoki Terada is an Editorial Board member of the *International Journal of Urology* and a co‐author of this article. To minimize bias, he was excluded from all editorial decision‐making related to the acceptance of this article for publication.

## Supporting information


**Figure S1.** A typical example of the bedtime (*X*) and mid‐wake time ($f\left(X\right)$) data for a week for one participant. The average of *X* was 21:35 (20:59–22:32), and the average of $f\left(X\right)$ was 59.9 ± 17.5 min. As a result, the slope $\frac{\mathrm{df}\left(X\right)}{dX}$ was −0.44, and the optimal bedtime was $\hat{X}$= 21:35–150 × −0.44 = 22:41.

**Figure S2.** Scatter plot of the changes in NUF and bedtime. The correlation between the changes in NUF (*X‐*axis) and the changes in bedtime (*Y*‐axis) in the intervention period (*n* = 24). The changes in NUF and bedtime were significantly correlated (*r* = −0.58, *p* = 0.003 in Spearman’s rank test). NUF, nocturnal urinary frequency.
