# Supplementary figures and images for: Adjustment to an Appropriate Bedtime Improves Nocturia in Older Adults: A Crossover Study
Source: Int J Urol. 2025 Apr 26;32(7):870–6. doi: 10.1111/iju.70068 (PMC12230910; doi:10.1111/iju.70068)

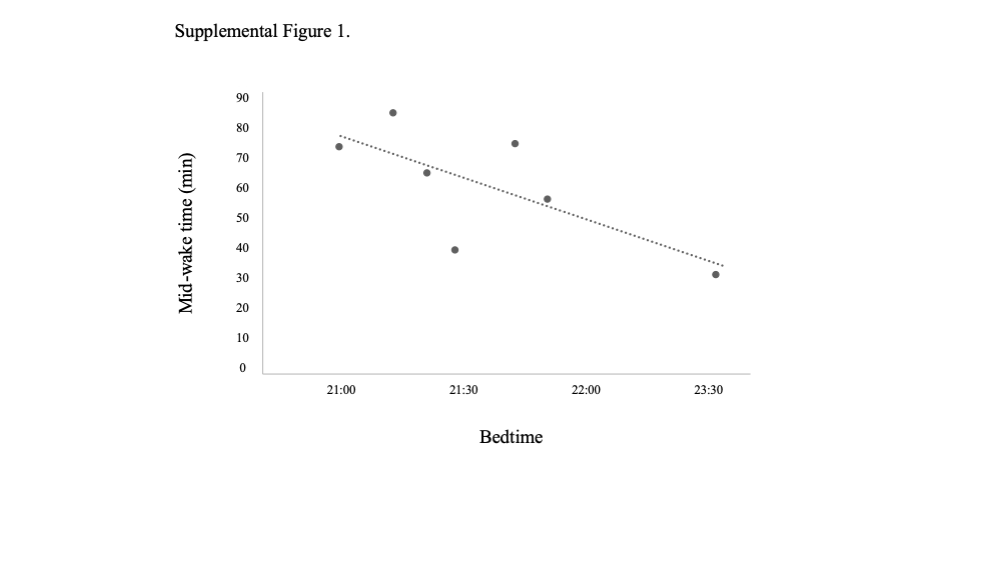

Supplement: Supplementary file 1 — Figure S1. A typical example of the bedtime (X) and mid‐wake time (fX) data for a week for one participant. The average of X was 21:35 (20:59–22:32), and the average of fX was 59.9 ± 17.5 min. As a result, the slope dfXdX was −0.44, and the optimal bedtime was X^= 21:35–150 × −0.44 = 22:41. [file IJU-32-870-s001.tiff]

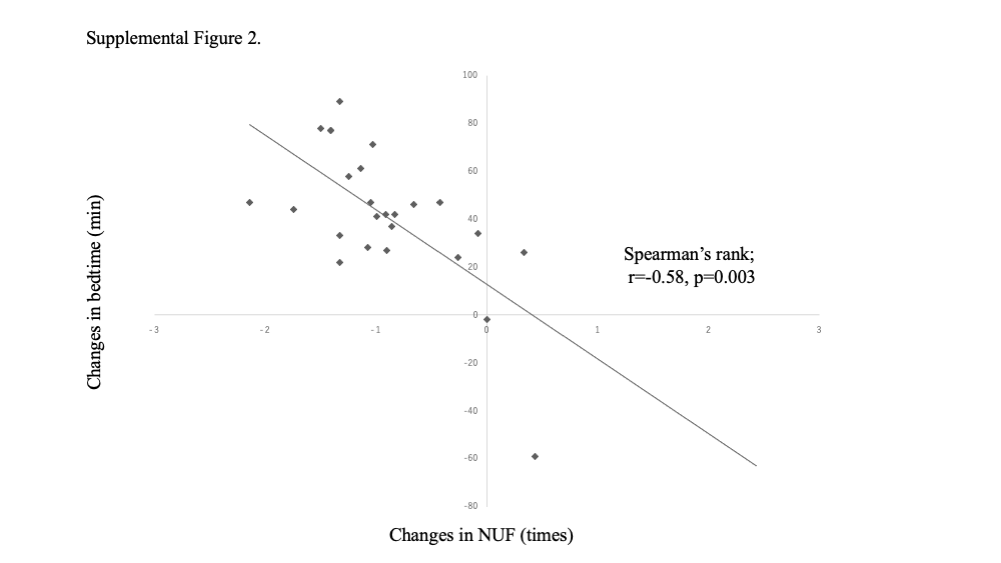

Supplement: Supplementary file 2 — Figure S2. Scatter plot of the changes in NUF and bedtime. The correlation between the changes in NUF (X‐axis) and the changes in bedtime (Y‐axis) in the intervention period (n = 24). The changes in NUF and bedtime were significantly correlated (r = −0.58, p = 0.003 in Spearman’s rank test). NUF, nocturnal urinary frequency. [file IJU-32-870-s002.tiff]
